# Heart failure in primary care: prevalence related to age and comorbidity

**DOI:** 10.1017/S1463423618000889

**Published:** 2019-07-29

**Authors:** Lieke Bosch, Patricia Assmann, Wim J. C. de Grauw, Bianca W. M. Schalk, Marion C. J. Biermans

**Affiliations:** Department of Primary and Community Care, Radboud University Medical Centre, Nijmegen, The Netherlands

**Keywords:** cohort study, comorbidities, diagnosis, heart failure, prevalence, primary health care

## Abstract

**Background:**

Diagnosing heart failure (HF) in primary care can be challenging, especially in elderly patients with comorbidities. Insight in the prevalence, age, comorbidity and routine practice of diagnosing HF in general practice may improve the process of diagnosing HF.

**Aim:**

To examine the prevalence of HF in relation to ageing and comorbidities, and routine practice of diagnosing HF in general practice.

**Methods:**

A retrospective cohort study was performed using data from electronic health records of 56 320 adult patients of 11 general practices. HF patients were compared with patients without HF using descriptive analyses and *χ* ^2^ tests. The following comorbidities were considered: chronic obstructive pulmonary disorder (COPD), diabetes mellitus (DM), hypertension, anaemia and renal function disorder (RFD). Separate analyses were performed for men and women.

**Findings:**

The point prevalence of HF was 1.2% (95% confidence interval 1.13–1.33) and increased with each age category from 0.04% (18–44 years) to 20.9% (⩾85 years). All studied comorbidities were significantly (*P*<0.001) more common in HF patients than in patients without HF: COPD (24.1% versus 3.1%), DM (34.7% versus 6.5%), hypertension (52.7% versus 16.0%), anaemia (10.9% versus 2.3%) and RFD (61.8% versus 7.5%). N-terminal pro-BNP (NT-proBNP) was recorded in 38.1% of HF patients.

**Conclusions:**

HF is highly associated with ageing and comorbidities. Diagnostic use of NT-proBNP in routine primary care seems underutilized. Instruction of GPs to determine NT-proBNP in patients suspected of HF is recommended, especially In elderly patients with comorbidities.

## Introduction

Heart failure (HF) affects an estimated 26 million people worldwide (Ambrosy *et al*., 2014) and this number is expected to be increasing due to the ageing population and improved survival following acute cardiac events (Hobbs *et al*., 2002; Bleumink *et al.*, 2004). Furthermore, the number of patients with risk factors for HF, in particular obesity and diabetes, is also increasing. Therefore, the prevalence of HF will probably increase in the near future (Danielsen *et al.*, 2017). Bleumink *et al*. (2004) and Hobbs *et al*. (2002) further appoint that HF is associated with a significant burden on quality of life, morbidity and mortality, with only 35% of patients surviving five years after diagnosis. Due to the poor prognosis and high morbidity, it causes high health-care-related costs. In 2011, the estimated costs were 940 million euro in the Netherlands, which was 1.1% of the total healthcare costs. Nearly 50% went to hospital care and 45% to nursing homes and home care (Rutten *et al.*, 2016).

HF is a complex and progressive syndrome in which the cardiac output is insufficient to meet the demands of the body due to an abnormality in cardiac structure or function, leading to a variety of symptoms and signs (Hoes *et al*., 2010; Yancy *et al*., 2013; Ponipowski *et al*., 2016). HF patients are frequently managed by general practitioners (GPs), which means every GP will encounter HF patients in their practice (Hoes *et al*., 1998). Diagnosing HF is difficult, because many of the symptoms are non-discriminating and, therefore, of limited diagnostic value (Remes *et al*., 1991; Mant *et al*., 2009). Moreover, HF is frequently associated with comorbidities, hampering clinical assessment. According to the Dutch HF guidelines, the main (physical) comorbidities associated with HF are chronic obstructive pulmonary disease (COPD), diabetes mellitus (DM), hypertension, anaemia and renal function disorder (RFD). These comorbidities may lead to or maintain HF (Hoes *et al*., 2010).

According to the current international and Dutch HF guidelines, GPs can use the plasma concentration of brain natriuretic peptide (BNP) and N-terminal pro-brain natriuretic peptide (NT-proBNP) as an initial diagnostic test (Hoes *et al*., 2010; Yancy *et al*., 2013; Ponipowski *et al*., 2016). Ponipowski *et al.* (2016) further recommend echocardiography to establish the diagnosis in patients with suspected HF. In the Netherlands, the GP fulfills a role as gatekeeper, which means that referrals to specialists are largely controlled by GPs. Until recently, Dutch GPs needed to refer patients with suspected HF to the cardiologist for echocardiography. Nowadays, they can order it directly.

Insight in the occurrence of HF in relation to age and comorbidity in routine general practice may improve the process of diagnosing HF. Therefore, the aim of our study is to examine the current prevalence of HF in relation to ageing, and the occurrence of the main comorbidities in patients with HF, separately for men and women as diagnosed by GPs in routine daily practice. In addition, we want to describe the process of diagnosing HF in routine primary care based on electronic health record (EHR) data. Insight in the routine diagnostic process may be helpful to improve care for this vulnerable group of patients.

## Methods

### Design and study population

We conducted a retrospective cohort study, using data of patients of eleven general practices participating in a Primary Care Practice Based Research Network, the Nijmeegs Monitoring Project (NMP, 2011). GPs and practice nurses of these general practices record data on care, disease and comorbidity. The data provided by the NMP are first encoded and subsequently delivered to the Department of Primary and Community Care in the Radboud University Medical Center (Radboudumc) Nijmegen, the Netherlands. Patients of NMP general practices are aware that their encoded data can be used in research by the Radboudumc, and are given the opportunity to object at any time (NMP, 2011).

Our study population consists of all adult patients (⩾18 years of age) that were signed up at the general practice at some point between 01/01/2010 and 31/12/2014. To answer our research questions, we created two groups. The first group, called the prevalence group (PG), consists of all patients that were present on 31/12/2014 to estimate the point prevalence. The second group, the diagnosis group (DG), consists of all adult patients (including the PG group).

### Heart failure, chronic comorbidities and clinical tests

HF is encoded using the International Classification of Primary Care (ICPC) version 1 (ICPC1) or version 2 (ICPC2) complemented with the International Classification of Diseases-10. These ICPC codes are used to register morbidities as well as test results during anamnesis (e.g., heart murmur). Patients were defined as having HF when the ICPC code started with K77. When HF was recorded more than once, we selected the first valid date of diagnosis, since we considered HF a chronic disease. This also applied to the included – chronic – comorbidities.

Demographic information included gender and age, which was categorized (18–44/45–54/55–64/65–74/75–84/⩾85). Since HF rarely occurs in patients younger than 44 years, the first category was aggregated. The other categories were chosen to show the (expected) correlation between higher age and the occurrence of HF. In the PG, the mean age was calculated on 31/12/2014. In the DG we included patients with and without HF, so it was not possible to calculate the mean age at the time of diagnosis. The mean age was therefore calculated on 01/01/2010.

The following chronic comorbidities were included: COPD, DM, hypertension, anaemia and RFD (Hoes *et al*., 2010). The ICPC codes used for these comorbidities are presented in Table 1. For RFD we additionally included the laboratory test for renal disease: estimated Glomerular Filtration Rate (eGFR). When eGFR <60 mL/min/1.73 m^2^ was present, the patient was added as a patient with RFD.

**Table 1:** Overview of included variables in diagnosis group (Dutch College of General Practitioners 2016)

	Measurement code(s)	Outcome	Coding ICPC1	ICPC2	ICD10
Comorbidities					
COPD	–	–	R95	R95	–
DM	–	–	T90.01 T90.02	T89 T90	E10 E11–E14
Hypertension	–	–	K85 K86 K87	K85 K86 K87	–
Anaemia	–	–	B80	B80	–
RFD	1919	Normal (⩾60 mL/min×1.73 m^2^)/reduced (<60 mL/min×1.73 m^2^)	U99.01	U99	N19
Symptoms					
Dyspnoea	1659	Yes/no/unclear	R02	R02	–
Orthopnoea	3010	Yes/no/unclear	–	–	–
Fatigue	3256	Absent/present/unclear	–	–	–
Anginal symptoms	1595	Yes/no/unclear	–	–	–
Signs					
Pulmonary crepitations	1868 2026	Normal/aberrant/unclear Crepitations/rhonchi/extended expirium/eliminated breathing sounds/remaining	–	–	–
Peripheral oedema	3007	Yes/no/unclear	K07	K07	–
Elevated central venous pressure	3000	Free text	–	–	–
Enlarged liver	3005	Free text	D96	D23	–
Ascites	–		D29.01	D29	R18
Widened ictus cordis	2062	Inside midclavicular line/outside midclavicular line/unclear	–	–	–
Heart murmur	2060 2061	Normal/aberrant/unclear Free text	K81	K81	–
Additional clinical tests					
BNP	1966	Normal (<10 pmol/L)/elevated (⩾10 pmol/L)	–	–	–
NT-proBNP	1968	Normal (<15 pmol/L)/elevated (⩾15 pmol/L)	–	–	–
Chest X-ray	2321	Free text	–	–	–
Electrocardiography	2202	Free text	–	–	–
Echocardiography	3001	Free text	–	–	–

ICPC=International Classification of Primary Care; ICD=International Classification of Diseases; COPD=chronic obstructive pulmonary disease; DM=diabetes mellitus; RFD=renal function disorder; BNP=brain natriuretic peptide; NT-proBNP=N-terminal pro-BNP.

Routine practice of the diagnostic procedure in general practice was described according to the three pillars in the algorithm from the HF guidelines: clinical symptoms in anamnesis, signs during physical examination and additional clinical tests (Hoes *et al*., 2010; Yancy *et al*., 2013; Ponipowski *et al*., 2016). This information was available in ICPC codes, measurements and/or free text (Table 1). We had no access to free text due to privacy regulations (Krabben, 2005). The measurements were only available from 2008 and onwards and comprised two elements: (1) whether the measurements were recorded or not and (2) the outcome of the measurement: suspected for HF or not. Of these, we selected the first outcomes of the symptoms and signs that were suspected for HF. This meant that an aberrant value overrode a normal value. This also applied to the outcomes of the additional test results: an elevated value overrode a normal value. The cut-off points used for BNP/NT-proBNP were 10 and 15 pmol/L, respectively (Hoes *et al*., 2010). ICPC codes and measurements recorded in <15 patients were not included.

### Statistical methods

The point prevalence in the PG was estimated on 31/12/2014. We estimated this in men and women separately, and categorized by age. In order to determine whether correction for clustering was necessary, we calculated the intracluster correlation coefficient, which was virtually zero (0,00000395). Therefore, we did not correct for clustering. The Wilson’s score interval was used to measure the 95% confidence interval (95% CI). In the DG, we determined the comorbidities in patients with and without HF and categorized by gender and age. A *χ* ^2^ test was used to analyze differences in comorbidities between patients with and without HF. A *P*-value <0.05 was considered statistically significant. We also used the DG to describe the process of diagnosing. As mentioned previously, all ICPC codes assigned in the past were available, but measurements were only available from 2008 and onwards. In addition, the current Dutch guideline for HF is from 2010. For these reasons, we composed two groups: no HF and HF diagnosed in 2010 or later. Finally, we analyzed the symptoms, signs and additional clinical tests for each group and the total group (including additionally HF before 2010). First, we determined whether ICPC codes were present or not and whether measurements were recorded or not. Second, we determined if the outcomes were suspected for HF or not in the HF group. Statistical Package for the Social Sciences version 22 was used for statistical analysis. Characteristics of the PG and the DG were provided using descriptive statistics.

## Results

### Point prevalence of heart failure

On 31/12/2014, 605 of the eligible patients in our PG (*n*=49 362) were diagnosed with HF. The overall point prevalence of HF in the adult population was 1.3% (95% CI: 1.14–1.42) in men and 1.2% (95% CI: 1.06–1.33) in women (Table 2). The point prevalence in men and women increased with each age category, starting with 0.04% (95% CI: 0.02–0.07) in patients younger than 44 years and increasing to 20.9% (95% CI: 17.96–24.08) in patients aged 85 years or older. Until 54 years, men and women showed comparable point prevalences. The point prevalence in men aged 55–64 years was 1.0% (95% CI: 0.75–1.38) and in women 0.6% (95% CI: 0.37–0.84). Similar results were found in patients aged 65–74 years: 2.9% (95% CI: 2.37–3.57) and 1.5% (95% CI: 1.15–2.03), respectively. In patients aged 85 years or older, the point prevalence was 21.8% (95% CI: 16.98–27.52) and 20.4% (95% CI: 16.87–24.36), respectively.

**Table 2:** Prevalence (%) of heart failure in the adult population (prevalence group)

	Men		Women		Total	
Age (years)	*n*	Prevalence % (95% CI)	*n*	Prevalence % (95% CI)	*n*	Prevalence % (95% CI)
18–44	4	0.04 (0.01–0.10)	4	0.04 (0.01–0.09)	8	0.04 (0.02–0.07)
45–54	18	0.4 (0.23–0.57)	16	0.3 (0.20–0.53)	34	0.3 (0.25–0.48)
55–64	41	1.0 (0.75–1.38)	23	0.6 (0.37–0.84)	64	0.8 (0.62–1.00)
65–74	88	2.9 (2.37–3.57)	46	1.5 (1.15–2.03)	134	2.2 (1.88–2.62)
75–84	108	8.2 (6.69–9.85)	116	7.9 (6.59–9.34)	224	8.0 (7.08–9.10)
⩾85	51	21.8 (16.98–27.52)	90	20.4 (16.87–24.36)	141	20.9 (17.96–24.08)
Total	310	1.3 (1.14–1.42)	295	1.2 (1.06–1.33)	605	1.2 (1.13–1.33)

CI=confidence interval.

### Comorbidities in heart failure patients

**Figure 1:**
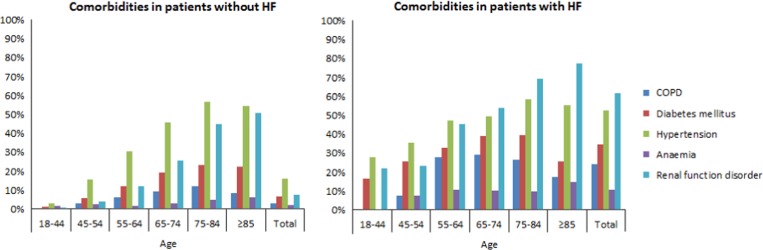
Bar graph of comorbidities in patients with and without heart failure per age category HF=heart failure; COPD=chronic obstructive pulmonary disease

From the DG, three patients were excluded for whom no date of diagnosis was known, leading to a total of 56 320 patients. Of this group, 55 224 patients had no HF and 525 patients were diagnosed with HF in 2010 or later. COPD (24.1%), DM (34.7%), hypertension (52.7%), anaemia (10.9%) and RFD (61.8%) were highly common in patients with HF (Table 3). These comorbidities were significantly lower in patients without HF (*P*<0.001). Similar results applied to both men and women (*P*<0.001). COPD was seen in 30.3% of men and in 18.0% of women with HF. The opposite applied to hypertension, which was seen in 57.2% of women and 47.9% of men with HF, and RFD, which was seen in 67.3% of women and 56.1% of men with HF. In addition, statistical significance was calculated for each age category, which is also shown in Table 3. The number of each comorbidity increased with each age category, with a peak around 65–74 or 75–84 years, and decreased in patients aged 85 years or older. Exceptions were RFD and anaemia, which had the highest numbers in patients aged 85 years or older. Moreover, anaemia was seen most in women aged 45–54 years without HF (4.4%) and in women aged 55–64 years with HF (13.7%). Figure 1 shows comorbidities in patients with and without HF per age category.

**Table 3:** Comorbidities in patients with and without heart failure (HF)

	Men (*n*=27 556)					Women (*n*=28 764)					Total (*n*=56 320)				
	No HF (*n*=27 019)		HF (*n*=537)			No HF (*n*=28 205)		HF (*n*=559)			No HF (*n*=55 224)		HF (*n*=1096)		
	*n*	%	*n*	%	*P*-value	*n*	%	*n*	%	*P*-value	*n*	%	*n*	%	*P*-value
COPD	**937**	**3.5**	**163**	**30.3**	**<0.001**	**801**	**2.8**	**101**	**18.0**	**<0.001**	**1738**	**3.1**	**264**	**24.1**	**<0.001**
18–44	64	0.5	0	0.0	>0.99	55	0.4	0	0.0	>0.99	119	0.4	0	0.0	>0.99
45–54	145	2.9	4	12.9	**0.012**	172	3.3	0	0.0	>0.99	317	3.1	4	7.8	0.074
55–64	260	6.1	24	29.6	**<0.001**	277	6.6	13	25.5	**<0.001**	537	6.3	37	28.0	**<0.001**
65–74	286	11.6	44	29.5	**<0.001**	175	6.9	29	28.4	**<0.001**	461	9.2	73	29.1	**<0.001**
75–84	162	16.8	72	35.8	**<0.001**	98	8.2	37	17.5	**<0.001**	260	12.0	109	26.4	**<0.001**
⩾85	20	12.0	19	28.8	**0.002**	24	6.9	22	13.3	**0.018**	44	8.6	41	17.7	**<0.001**
DM	**1920**	**7.1**	**181**	**33.6**	**<0.001**	**1660**	**5.9**	**199**	**35.5**	**<0.001**	**3580**	**6.5**	**380**	**34.7**	**<0.001**
18–44	200	1.4	2	22.2	**0.007**	192	1.3	1	11.1	0.11	392	1.4	3	16.7	**0.002**
45–54	341	6.8	11	35.5	**<0.001**	236	4.5	2	10.0	0.23	577	5.6	13	25.5	**<0.001**
55–64	601	14.0	24	29.6	**<0.001**	414	9.8	19	37.3	**<0.001**	1015	11.9	43	32.6	**<0.001**
65–74	505	20.5	58	38.9	**<0.001**	470	18.6	40	39.2	**<0.001**	975	19.5	98	39.0	**<0.001**
75–84	234	24.2	72	35.8	**0.001**	273	22.8	92	43.4	**<0.001**	507	23.5	164	39.7	**<0.001**
⩾85	39	23.5	14	21.2	0.71	75	21.7	45	27.3	0.16	114	22.3	59	25.5	0.33
Hypertension	**4022**	**14.9**	**257**	**47.9**	**<0.001**	**4822**	**17.1**	**321**	**57.2**	**<0.001**	**8844**	**16.0**	**578**	**52.7**	**<0.001**
18–44	362	2.6	2	22.2	0.21	491	3.3	3	33.3	**0.003**	853	3.0	5	27.8	**<0.001**
45–54	745	14.8	11	35.5	**0.004**	848	16.3	7	35.0	**0.033**	1593	15.5	18	35.3	**<0.001**
55–64	1305	30.4	41	50.6	**<0.001**	1294	30.6	21	41.2	0.073	2599	30.5	62	47.0	**<0.001**
65–74	1053	42.8	69	46.3	0.39	1241	49.0	55	53.9	0.33	2294	45.9	124	49.4	0.28
75–84	487	50.5	105	52.2	0.65	739	61.8	136	64.2	0.51	1226	56.7	241	58.4	0.54
⩾85	70	42.2	29	43.9	0.81	209	60.4	99	60.0	0.93	279	54.5	128	55.4	0.82
Anaemia	**241**	**0.9**	**56**	**10.4**	**<0.001**	**1024**	**3.6**	**63**	**11.2**	**<0.001**	**1265**	**2.3**	**119**	**10.9**	**<0.001**
18–44	27	0.2	0	0.0	>0.99	534	3.6	0	0.0	>0.99	561	1.9	0	0.0	>0.99
45–54	41	0.8	2	6.5	**0.028**	231	4.4	2	10.0	0.22	272	2.7	4	7.8	**0.047**
55–64	44	1.0	7	8.6	**<0.001**	108	2.6	7	13.7	**<0.001**	152	1.8	14	10.6	**<0.001**
65–74	74	3.0	16	10.7	**<0.001**	71	2.8	10	9.8	**0.001**	145	2.9	26	10.4	**<0.001**
75–84	42	4.4	21	10.4	**0.001**	62	5.2	20	9.4	0.15	104	4.8	41	9.9	**<0.001**
⩾85	13	7.8	10	15.2	0.09	18	5.2	24	14.5	**<0.001**	31	6.1	34	14.7	**<0.001**
RFD	**1704**	**6.3**	**301**	**56.1**	**<0.001**	**2464**	**8.7**	**376**	**67.3**	**<0.001**	**4168**	**7.5**	**677**	**61.8**	**<0.001**
18–44	92	0.7	2	22.2	**0.002**	113	0.8	2	22.2	**0.002**	205	0.7	4	22.2	**<0.001**
45–54	150	3.0	7	22.6	**<0.001**	277	5.3	5	25.0	**0.003**	427	4.2	12	23.5	**<0.001**
55–64	416	9.7	35	43.2	**<0.001**	596	14.1	25	49.0	**<0.001**	1012	11.9	60	45.5	**<0.001**
65–74	565	22.9	71	47.7	**<0.001**	724	28.6	64	62.7	**<0.001**	1289	25.8	135	53.8	**<0.001**
75–84	398	41.2	134	66.7	**<0.001**	576	48.2	153	72.2	**<0.001**	974	45.1	287	69.5	**<0.001**
⩾85	83	50.0	52	78.8	**<0.001**	178	51.4	127	77.0	**<0.001**	261	51.0	179	77.5	**<0.001**

COPD=chronic obstructive pulmonary disease; DM=diabetes mellitus; RFD=renal function disorder. Bold P-values are statistically significant.

### Process of diagnosing heart failure

Of all symptoms, the ICPC code for dyspnoea was recorded in 21.9% of patients with HF and in 3.4% of patients without HF (Table 4). Anginal symptoms were recorded in 28.2 and 9.6%, respectively. Of the signs, the ICPC code for peripheral oedema was recorded in 27.2% of patients with HF and in 3.2% of patients without HF. Of the additional tests, NT-proBNP was recorded in 38.1% of patients with HF and in 3.5% of patients without HF. Anginal symptoms and auscultation of the lungs were suspected for HF in 15.5 and 54.8% of the cases, respectively (not in Table). NT-proBNP was elevated in 93.5% of the cases (not in Table).

**Table 4:** Recorded symptoms, signs and additional tests in patients with and without heart failure (HF)

	No HF		HF diagnosed in 2010 or later		Total (diagnosis group)^a^	
	*n*=55 224	98.1%	*n*=525	0.9%	*n*=56 320	100%
Symptoms						
Dyspnoea ICPC	1870	3.4	115	21.9	2071	3.7
Dyspnoea anamnesis	423	0.8	11	2.1	444	0.8
Anginal symptoms	5294	9.6	148	28.2	5576	9.9
Signs						
Auscultation lungs	1166	2.1	31	5.9	1227	2.2
Abnormalities auscultation lungs	179	0.3	17	3.2	206	0.4
Peripheral oedema ICPC	1782	3.2	143	27.2	2034	3.6
Peripheral oedema physical examination	56	0.1	2	0.4	58	0.1
Auscultation heart	363	0.7	8	1.5	381	0.7
Abnormalities auscultation heart	23	0.04	1	0.2	25	0.04
Heart murmur ICPC	363	0.7	6	1.1	372	0.7
Additional tests						
BNP	6	0.01	0	0.0	16	0.03
NT-proBNP	1934	3.5	200	38.1	2365	4.2

ICPC=International Classification of Primary Care; BNP=brain natriuretic peptide; NT-proBNP=N-terminal pro-BNP. Not/seldom recorded: symptoms: orthopnoea, fatigue, signs: elevated central venous pressure, enlarged liver, ascites, widened ictus cordis, additional tests: echocardiography, electrocardiography, chest X-ray.

a This group included no HF (*n*=55 224), HF diagnosed in 2010 or later (*n*=525) and HF before 2010 (*n*=571).

## Discussion

### Prevalence, age and comorbidities

This retrospective cohort study showed an overall point prevalence of HF of 1.2% in the adult population. Prevalence increased with each age category from 0.04% in patients younger than 44 years to 20.9% in patients aged 85 years or older. Comorbidities, COPD, DM, hypertension, anaemia and RFD, were significantly more common in patients with HF than in patients without HF. The symptoms and signs dyspnoea, anginal symptoms and peripheral oedema were reported most frequently in the process of diagnosing HF.

### NT-proBNP

NT-proBNP was recorded in only 38.1% of HF patients, although it is recommended in the HF guidelines as a test to assist GPs to rule out HF. Especially in elderly patients with comorbidities, such as COPD, HF patients may be overdiagnosed due to overlap in symptoms and signs (Brenner *et al*., 2013). In these patients, NT-proBNP would be useful to prevent overdiagnosing HF.

### Comparison with existing literature

The prevalence rates found in our study are not comparable to those presented in other studies, due to differences in region, population (not primary care) and definitions/methods to assess HF. Most studies estimated the prevalence of HF by conducting a population-based study in which patients underwent diagnostic work-up and sometimes an expert panel was used to make the final diagnosis. Moreover, these studies only included patients older than 45–55 years, which led to higher overall prevalence rates (Mosterd *et al*., 1999; Daamen *et al*., 2015). However, Engelfriet *et al.* (2012) and Van Baal *et al.* (2010) did conduct a comparable study to estimate the prevalence rates of HF in the Netherlands. They found comparable overall prevalence rates, but lower rates (between 11 and 16%), in patients aged 85 years or older. These differences can possibly be explained by the ageing of the population in the Netherlands. Our findings regarding the high number of comorbidities in HF patients were consistent with other studies (Van Deursen *et al*., 2014). Although common risk factors are likely to contribute, HF itself might cause other comorbidities, and treatment of HF may have a negative impact, for example on renal function. Consequently, HF, often complicated by comorbidities, has started to put a great burden on the GP in recent years, which can be expected to increase even more.

In our study, the selected variables to represent symptoms, signs and additional tests were barely recorded. It is plausible to assume that these variables are not recorded in the way they were extracted because GPs use free text in patients’ medical files to describe data instead of coding them. A study of Vijayakrishnan *et al.* (2014), performed in the USA, used data extracted from free text and showed much higher percentages for documented symptoms and signs in primary care. As mentioned before, we had no access to free text in the EHR in our database which is in accordance with the guidelines of the Dutch data protection authorities (Krabben, 2005). In addition, diagnostic tests performed in secondary care, are not included in the primary care EHR. Confirmation by echocardiography is mandatory to diagnose HF (Hoes *et al*., 2010; Yancy *et al*., 2013; Ponipowski *et al*., 2016). Valk *et al.* (2016) performed a study in which turned out that more than one-third of patients labelled with HF in primary care might not have HF. In this study, in case patients labelled with HF had not yet undergone echocardiography, their GP was recommended to refer for an echocardiography to confirm the diagnosis. This shows the importance of echocardiography in the process of diagnosing HF. Unfortunately no data of echocardiography were available in our study, because this is recorded in free text.

### Strengths and limitations

Some limitations of our study should be considered. First, we made no distinction between acute and chronic HF, therefore we used the lowest cut-off point of NT-proBNP. This did not alter our findings since, according to the guidelines, the diagnosis of acute HF is primarily based on anamnesis and physical examination (Hoes *et al*., 2010; Yancy *et al*., 2013; Ponipowski *et al*., 2016), and we already included these in our study. Another limitation is that we might have missed recordings of diagnostic tests for HF since these tests could be performed in secondary care. It is estimated that about 50% of patients with HF are referred to a cardiologist (RIVM, 2018). Furthermore, we could not use free text to study symptoms, signs and additional clinical tests and therefore we could not study echocardiographic assessment unfortunately. Besides, recall or recording bias might have arisen in this study concerning the numbers for dyspnoea, anginal symptoms and peripheral oedema. However, this does not apply to BNP and NT-proBNP as all laboratory values that are ordered by the GP are added automatically to the EHR. Finally, our conclusions regarding the relation between HF and comorbidities are not based on specific age categories. However, when looking at HF and comorbidities within the different age categories, the majority remains statistically significant. For example, RFD shows statistical significance in every age category.

A strength of our study is that it describes routine care for HF patients in general practice, where most HF patients are diagnosed and managed. Furthermore, we used data from general practices which are affiliated with the Radboudumc and have been shown to have very accurate ways of registration (Van der Wel *et al*., 2008). Another strength of our study is that we believe our study population to be representative for the overall population and therefore, our study results to be generalizable to patients with and without HF in the general adult population.

### Implications for practice

HF is highly associated with ageing and comorbidities, which makes the process of diagnosing HF in our ageing population more challenging. The complexity of this syndrome requires more comorbidity-specific recommendations in the existing guidelines, especially for the combination of HF and COPD or RFD. Our results show that symptoms and signs of HF were infrequently recorded and that additional tests were requested in 38% of the patients. This is partly explained by the fact that the diagnostic process may have been performed in secondary care, which was not included in our database. Also, these findings reflect a combination of the recording discipline of the GPs and the actual performance on diagnostic procedures. Despite these caveats, our study indicates that the process of diagnosing HF shows room for improvement. In particular, diagnostic use of NT-proBNP in routine primary care seems underutilized. Preliminary qualitative analysis among GPs from our research institute, suggests that (lack of) knowledge of the HF guidelines determines the (lack of) of measurement of NT-proBNP. Instruction of GPs to determine NT-proBNP in patients suspected of HF is recommended, especially in elderly patients with comorbidities.

## Declaration

Funding: none. Ethical approval: this study was performed according to the code of conduct for health research, which has been approved by the data protection authorities for conformity with the applicable Dutch privacy legislation. Competing interests: the authors have declared no competing interests.

## Authors’ Contribution

Design of the study; L.B., P.A., B.W.M.S., M.C.J.B. Data collection; L.B., B.W.M.S. Data entry; L..B., B.W.M.S. Data analysis; L.B., B.W.M.S., M.C.J.B. Interpretation of the data; all authors. Preparation of manuscript draft; all authors. All authors provided intellectual content and approved the final version. L.B. is the guarantor of the study.
